# A *SACS* deletion variant in Great Pyrenees dogs causes autosomal recessive neuronal degeneration

**DOI:** 10.1007/s00439-023-02599-1

**Published:** 2023-09-27

**Authors:** Kari J. Ekenstedt, Katie M. Minor, G. Diane Shelton, James J. Hammond, Andrew D. Miller, Susan M. Taylor, Yanyun Huang, James R. Mickelson

**Affiliations:** 1 Department of Basic Medical Sciences, College of Veterinary Medicine, Purdue University, Lynn Hall, 625 Harrison Street, West Lafayette, IN 47907 USA; 2 Department of Veterinary and Biomedical Sciences, College of Veterinary Medicine, University of Minnesota, Saint Paul, MN 55108 USA; 3 Department of Pathology, School of Medicine, University of California San Diego, La Jolla, CA 92093 USA; 4 Department of Neurology, Pieper Memorial Veterinary Center, Middletown, CT 06457 USA; 5 Section of Anatomic Pathology, Department of Population Medicine and Diagnostic Sciences, College of Veterinary Medicine, Cornell University, Ithaca, NY 14853 USA; 6 Department of Small Animal Clinical Sciences, Western College of Veterinary Medicine, University of Saskatchewan, Saskatoon, SK S7N 5B4 Canada; 7 Prairie Diagnostic Services, Inc., Saskatoon, SK S7N 5B4 Canada

## Abstract

ARSACS (autosomal recessive spastic ataxia of Charlevoix-Saguenay) is a human neurological disorder characterized by progressive cerebellar ataxia and peripheral neuropathy. A recently recognized disorder in Great Pyrenees dogs is similarly characterized by widespread central nervous system degeneration leading to progressive cerebellar ataxia and spasticity, combined with peripheral neuropathy. Onset of clinical signs occurred in puppies as young as 4 months of age, with slow progression over several years. A multi-generation pedigree suggested an autosomal recessive mode of inheritance. Histopathology revealed consistent cerebellar Purkinje cell degeneration, neuronal degeneration in brainstem nuclei, widespread spinal cord white matter degeneration, ganglion cell degeneration, inappropriately thin myelin sheaths or fully demyelinated peripheral nerve fibers, and normal or only mild patterns of denervation atrophy in skeletal muscles. Genome-wide single nucleotide polymorphism (SNP) genotype data was collected from 6 cases and 26 controls, where homozygosity mapping identified a 3.3 Mb region on CFA25 in which all cases were homozygous and all controls were either heterozygous or homozygous for alternate haplotypes. This region tagged the *SACS* gene where variants are known to cause ARSACS. Sanger sequencing of *SACS* in affected dogs identified a 4 bp deletion that causes a frame shift and truncates 343 amino acids from the C terminus of the encoded sacsin protein (p.Val4244AlafsTer32). Our clinical and histopathological descriptions of this canine disorder contribute to the description of human ARSACS and represents the first naturally occurring large animal model of this disorder.

**Supplementary Information:**

The online version contains supplementary material available at 10.1007/s00439-023-02599-1.

## Introduction

Almost 400 Mendelian inherited traits and disorders have been described in the domestic dog, *Canis lupus familiaris* and reported in the Online Mendelian Inheritance in Animals database (https://omia.org/home/). A likely causal variant is known for over 330 of these (accessed April 2023, https://omia.org/home/), and variants associated with hereditary ataxias (cerebellar ataxia, spinocerebellar ataxia, and cerebellar abiotrophy or cerebellar cortical degeneration) have been described in several breeds (Zeng et al. 2011; Kyöstilä et al. 2012; Forman et al. 2012, 2013, 2015; Agler et al. 2014; Gilliam et al. 2014; Rohdin et al. 2015; Gerber et al. 2015; Fenn et al. 2016; Mauri et al. 2017; Letko et al. 2019; Jenkins et al. 2020; Christen et al. 2021; Abitbol et al. 2022; Stee et al. 2023; Wade et al. 2022). Many of these closely recapitulate human conditions resulting from mutations in the homologous genes, providing large animal models for therapeutic and other investigations (Gilliam et al. 2014; Gerber et al. 2015; Rohdin et al. 2015; Mauri et al. 2017; Letko et al. 2019; Abitbol et al. 2022).

Autosomal recessive spastic ataxia of Charlevoix-Saguenay or ARSACS (Online Mendelian Inheritance in Man database [OMIM] #270,550) is an inherited ataxia in humans. ARSACS presents as an early-onset, slowly progressive neurodegenerative disorder characterized classically by a triad of symptoms: peripheral neuropathy with distal muscle wasting and weakness, a progressive cerebellar syndrome of ataxia, and lower limb spasticity (Vermeer et al. 2008); the original disease description also included hypermyelinated retinal nerve fibers (Bouchard et al. 1978; Takiyama 2007), although these can be more accurately described as hypertrophy of the retinal nerve fiber layer (RNFL) (Garcia-Martin et al. 2013a, b). ARSACS was first described in the Charlevoix-Saguenay region in Quebec, Canada, in the 1970s (Bouchard et al. 1978), where two founder mutations in the *SACS* gene (OMIM #604,490) were ultimately identified (Engert et al. 2000). The cohesive clinical picture in this group included early-onset, slowly progressive lower limb spasticity, truncal ataxia, and a mixed sensorimotor neuropathy that lead to distal muscle wasting, dysarthria, and nystagmus (Bouchard et al. 1978; Richter et al. 2004; Verhoeven et al. 2012). Since that time at least 350 additional disease-associated variants have been identified worldwide in the *SACS* gene (Gomez 2004; Stenson et al. 2017) (accessed March 2023). Increased description of *SACS* variants has been accompanied by reports of “atypical” clinical ARSACS cases that may not express the classical characteristics (e.g., without peripheral neuropathy, later onset of symptoms, etc.) (Baets et al. 2010; Synofzik et al. 2013), and/or may have additional abnormalities (e.g., intellectual disability, cognitive decline, epilepsy, etc.) (Takiyama 2007; Nascimento et al. 2016; Ali et al. 2016). Atypical patients with *SACS* variants may not have the RNFL hypertrophy (El Euch-Fayache et al. 2003; Criscuolo et al. 2004; Hara et al. 2005). While knockout mouse models exist (Girard et al. 2012; Larivière et al. 2015), and these accurately recapitulate classical human ARSACS, to date, no naturally occurring large animal model has been reported. Here, we provide extensive clinical and pathological descriptions, as well as molecular genetic characterization, of a novel spontaneous canine genetic model of *SACS*-associated neuronal degeneration.

## Materials and methods

### Ethics statement and sample collection and processing

Discovery group (*n* = 32) Great Pyrenees samples were obtained by solicitation through motivated breeders and owners. The follow-up Great Pyrenees samples were obtained via the Orthopedic Foundation for Animals (OFA) Canine Health Information Center (CHIC) biobank (after voluntary owner sample banking) or were submitted to our laboratory for subsequent genetic testing. Written consent was obtained from all dog owners. DNA was extracted from EDTA-blood or cheek swabs using standard methods. Medical histories were obtained for affected dogs only; control dogs were over 1 year of age with no owner-reported problems. The University of Minnesota’s Institutional Animal Care and Use Committee (IACUC) approved this study (#1503-32413A). Postmortem examinations were conducted on seven affected dogs to acquire pathological specimens; these were performed with the owners’ permission after humane euthanasia, or, in one case, after natural death. For all autopsied dogs, euthanasia was elected by the owner due to progression of disease, under guidance from the attending veterinarian. No dogs were euthanized directly for purposes of this study.

### Clinical examinations

Affected dogs (*n* = 8) were owned as pets, and were examined and treated under the care of both their general practice veterinarians and board-certified veterinary neurologists. Diagnostic testing of affected dogs variably included the following modalities, in addition to standard bloodwork: MRI, CT, motor nerve conduction velocity testing, and diagnostic peripheral nerve and muscle biopsies. Two affected dogs received standard ophthalmic examinations from a board-certified veterinary ophthalmologist, including fundoscopic exam; optical coherence tomography (OCT) was not performed.

### Pathological examinations

Elective autopsies were performed on seven affected dogs and included collection of both central nervous system and peripheral nerve tissues. The cerebrum, thalamus, cerebellum, and brainstem, and sections from each region of the spinal cord (cervical, thoracic, lumbar) were collected into 10% neutral buffered formalin and paraffin embedded. Additional samples were collected from all major organs and processed similarly. Sciatic, femoral, common peroneal, and ulnar nerves, and dorsal and ventral L5 nerve roots including portions of ganglia were fixed in 2.5% glutaraldehyde and embedded in araldite resin as previously described (Shelton et al. 2003). Paraffin sections were routinely processed and stained with hematoxylin and eosin, and resin Sects. (1 µm) stained with toluidine blue or paraphenylenediamine for light microscopy. For ophthalmological samples, one globe was immersed in Bouin’s solution or Davidson’s solution and one globe immersed in glutaraldehyde.

### Genome-wide association mapping and haplotype analysis

**Fig. 1:**
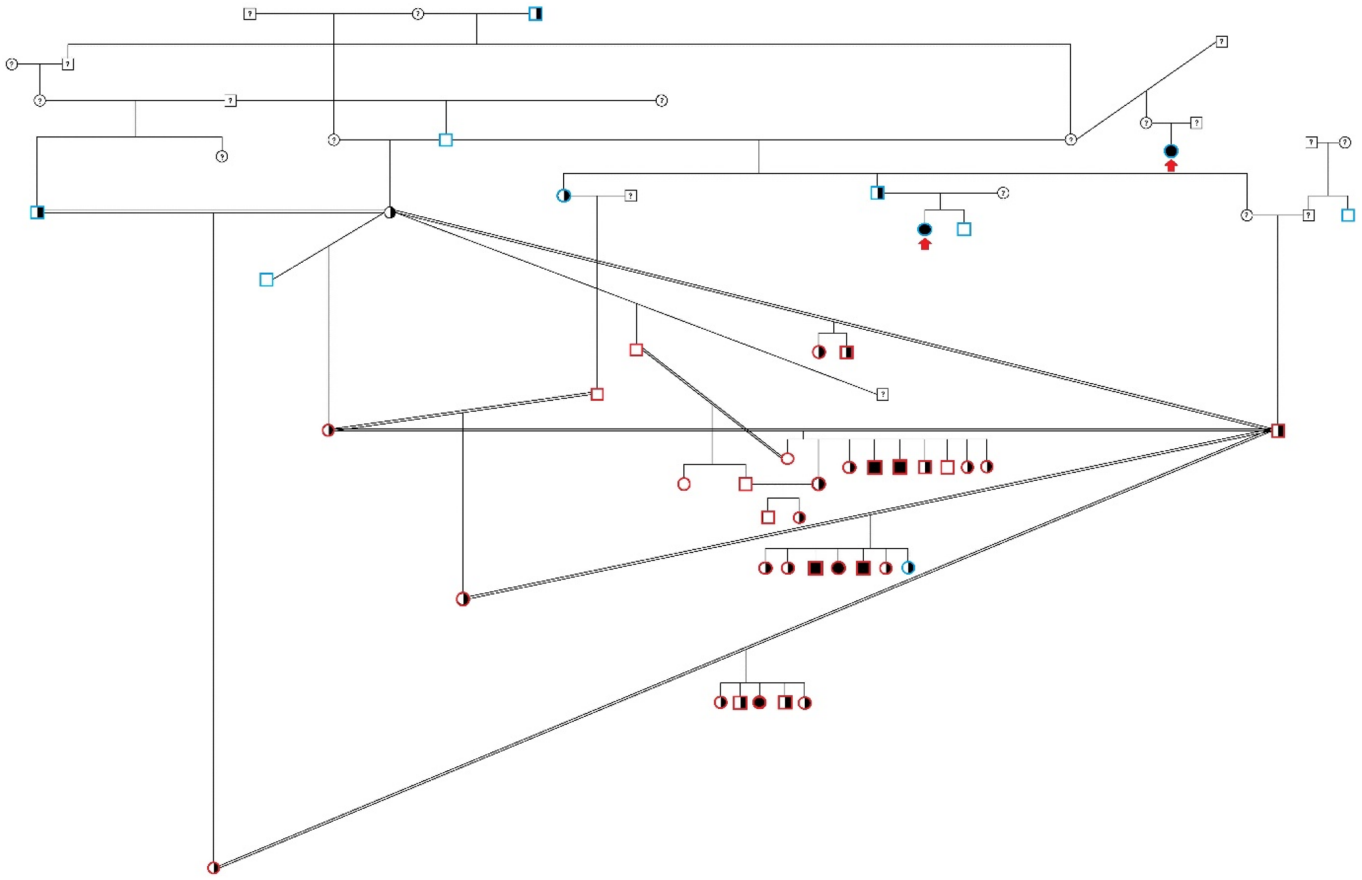
Pedigree of Affected Great Pyrenees family. Discovery group (*n* = 32, those with red rim) and extended family, including two subsequently identified cases (red arrows). All affected dogs can be traced to a common ancestor (not shown). The pedigree strongly suggests a recessive mode of inheritance. Males are indicated with squares, females with circles. Double bars indicate consanguineous matings. Filled symbols indicate affected dogs, all of whom were homozygous for the *SACS* deletion. Half-filled symbols indicate dogs that were heterozygous for the *SACS* deletion (carriers); all of these dogs are phenotypically normal. Open symbols indicate dogs that were homozygous for the reference allele. Blue rim indicates the dog was tested at a later time point (not in discovery group). Dogs not genetically tested are indicated with “?”, although some of their genotypes can be inferred

The discovery group consisted of 32 dogs (those with red rim, Fig. 1), including 6 affected (2 females, 4 males) and 26 controls (16 females, 10 males) that were genotyped for 173,000 SNPs on Illumina CanineHD 173 K BeadChips (Illumina; San Diego, CA). Data were analyzed in PLINK v1.9 (Purcell et al. 2007). Standard pruning parameters were used, with 90% cut-offs for genotyping per dog and per SNP, and 5% cut-off for minor allele frequency, leaving a final data set consisting of 70,830 SNPs. Standard chi-square association analysis (giving a genomic inflation factor of 1.2) and the --model test (a modified homozygosity mapping approach) were conducted in PLINK. The --fisher command was added to generate p-values. The --model test, in addition to the basic allele test, conducts four more tests of association: (1) Cochran-Armitage Trend test; (2) Genotypic (2 *df*) test; (3) Dominant gene action (1 *df*) test; and 4) Recessive gene action (1 *df*) test. Haplotype reconstruction was performed using fastPHASE (Scheet and Stephens 2006).

### Sequencing and genetic test development

The canine sequence of the positional and functional candidate *SACS* gene was obtained from Ensembl (www.ensembl.org) using the Basenji build (ENSCAFT00030020331.1) (CanFam3.1 and CanFam4 are annotated incorrectly for this gene), and aligned to the human sequence to verify annotation using MultAlin (http://multalin.toulouse.inra.fr/multalin/multalin.html) (Corpet 1988). PCR primers (Supplemental Table S1) were designed using Primer3 (http://biotools.umassmed.edu/bioapps/primer3_www.cgi) (Untergasser et al. 2012) for all canine *SACS* exons, their flanking intronic sequences, and the 5’ untranslated region (the 3’ UTR is not yet annotated, and follow-up sequencing was not performed due to discovery of the deletion reported below). Standard PCR conditions were used to amplify DNA segments from an affected dog. Amplicons were then subjected to Sanger sequencing, and sequences were visualized in Sequencher (v.5.1, Gene Codes Corp., Ann Arbor, MI).

Primers for an allele-specific PCR genotyping assay for the discovered associated *SACS* deletion variant are listed in Supplemental Table S2; each reaction includes one pair of the *SACS* allele-specific primers and an internal control pair of primers (designated LPN1). Each dog was tested twice, once with each *SACS* reverse primer. PCR products were visualized on a 2% agarose gel to determine the genotype (Supplemental Fig. 1).

### Post hoc linkage mapping and heritability calculations

A retrospective LOD (logarithm of the odds) score was calculated using each dog’s *SACS* deletion genotype and the known pedigree relationships (Fig. 1). Two-point autosomal linkage analysis was conducted using the “Linkage Analysis Package” in MLink (version 5.1) (Cottingham Jr et al. 1993), using 26 controls and 8 cases (6 original cases, plus two subsequently identified cases, shown with red arrows in Fig. 1; these two dogs had nearly identical clinical histories and disease courses, and both subsequently tested homozygous for the identified deletion). Heritability was calculated using the airemlf90 program within the BLUPF90 software package family (Misztal et al. 2002) using pedigree and phenotype information from all 65 available dogs in the pedigree (Fig. 1) and the Illumina CanineHD 173 K SNP genotypes from 32 dogs (those with red rim in Fig. 1).

### Screening of additional Great Pyrenees and dogs of other breeds

Great Pyrenees DNAs (*n* = 133) were obtained via the OFA CHIC DNA repository for an initial, presumably unbiased, study to estimate the *SACS* deletion allele frequency in the breed. Three of these dogs, one of each genotype, happened to overlap with dogs in the discovery cohort, otherwise, detailed phenotypic information was not available. Subsequently, a larger comprehensive cohort of Great Pyrenees (*n* = 972, including the discovery cohort, the population study, and all samples subsequently submitted for genetic testing) was also used to calculate the deletion allele frequency, with the understanding that this latter group represents some ascertainment bias. Publicly available variant call files (VCFs) generated via whole-genome sequencing (WGS), representing more than 1300 dogs from 126 diverse breeds (Jagannathan et al. 2019; Plassais et al. 2019), were searched to determine if the deletion variant was private to the Great Pyrenees breed.

## Results

### Disease description: clinical

All affected dogs were closely related (Fig. 1), and could be traced to a common ancestor (not shown on pedigree). This, together with visual assessment of the pedigree, strongly suggested a recessive mode of inheritance. Puppies appeared normal until 3–5 months of age. The first indications of disease include clumsiness, uncoordinated behavior, and having difficulty walking on slippery surfaces. Affected puppies were reluctant to climb or descend stairs and often looked for fences, walls, or even an owner to lean against while walking; they preferentially chose to lie down when that option was available (e.g., when eating). Clinical signs were slowly and insidiously progressive, with periods of apparent plateau. Signs were most consistent with cerebellar dysfunction, but dogs also displayed lower motor weakness in the distribution of the sciatic nerves. At younger ages, the cerebellar signs predominated; however, as the disease progressed, neuromuscular weakness was most pronounced. Clinical signs did not worsen with exercise, unlike other types of neuromuscular weakness.

Clinical signs were noticeable in all affected dogs before 2 years of age, with most developing subtle cerebellar ataxia before 1 year of age. Gait evaluation abnormalities included: (1) difficulty and eventual inability to ascend or descend stairs, (2) truncal sway, (3) pelvic limb base-wide stance, both when standing and when ambulating, (4) bunny-hopping in the pelvic limbs, (5) cerebellar ataxia, and (6) excessive coxofemoral flexion during the protraction phase of gait. Muscle atrophy developed in all limbs over time, although atrophy of thoracic limbs lagged behind that of the pelvic limbs. Spinal reflexes, including patellar and pelvic limb withdrawal reflexes, were progressively diminished. Eventual disease progression resulted in patients being unable to walk without assistance in the pelvic limbs. Most dogs were euthanized by 4–7 years of age, but one as young as 1.5 years.

No changes to mentation were present, either on neurological exams or reported by owners. Postural reactions were exaggerated in cases where cerebellar dysfunction was most significant. Cranial nerve functions were normal. Mild atrophy of temporalis and masseter muscles occurred with age. Two female cases (euthanized at 4.5 and 7.2 years) developed progressive head tremors, initially very fine and progressing to both resting and intentional head tremors. A third affected female (euthanized at 1.5 years) was reported free of head tremors, however, this may reflect a difference due to disease progression with age or the variable contribution of cerebellar dysfunction. Vocalization changed in at least two dogs, presumably due to recurrent laryngeal nerve involvement.

Two cases (one male, one female) were examined by a veterinary ophthalmologist at 2.5 years of age. There was no indication of excess myelination or “yellow streaks” (the RNFL hypertrophy seen in classic human ARSACS cases) on fundoscopic examination. The female did have linear translucent opacities at the lateral edges of the nontapetal area which appeared to be vitreal in nature. She also had very subtle nystagmus, which manifested when she was trying to focus vision on a finer scale (e.g., a fly on the floor). Affected dogs maintained normal vision. No evidence of resting or positional nystagmus was present on neurologic evaluations.

Screening bloodwork, when performed, including CBC, chemistry profile, ACTH stim, and thyroid levels, and urinalysis, were all within normal limits. Serum and CSF tests for *Neospora* and canine distemper virus antibodies, respectively, were negative. Electromyography performed on one affected dog revealed no abnormal spontaneous discharges in the appendicular musculature. Motor nerve conduction velocities measured on this dog were slowed (34 m/s in peroneal nerve, 35 m/s in ulnar nerve, reference > 50 m/s for both nerves). For both nerves, compound muscle action potentials amplitudes were within the reference range, with polyphasia observed only at the carpus.

**Fig. 2:**
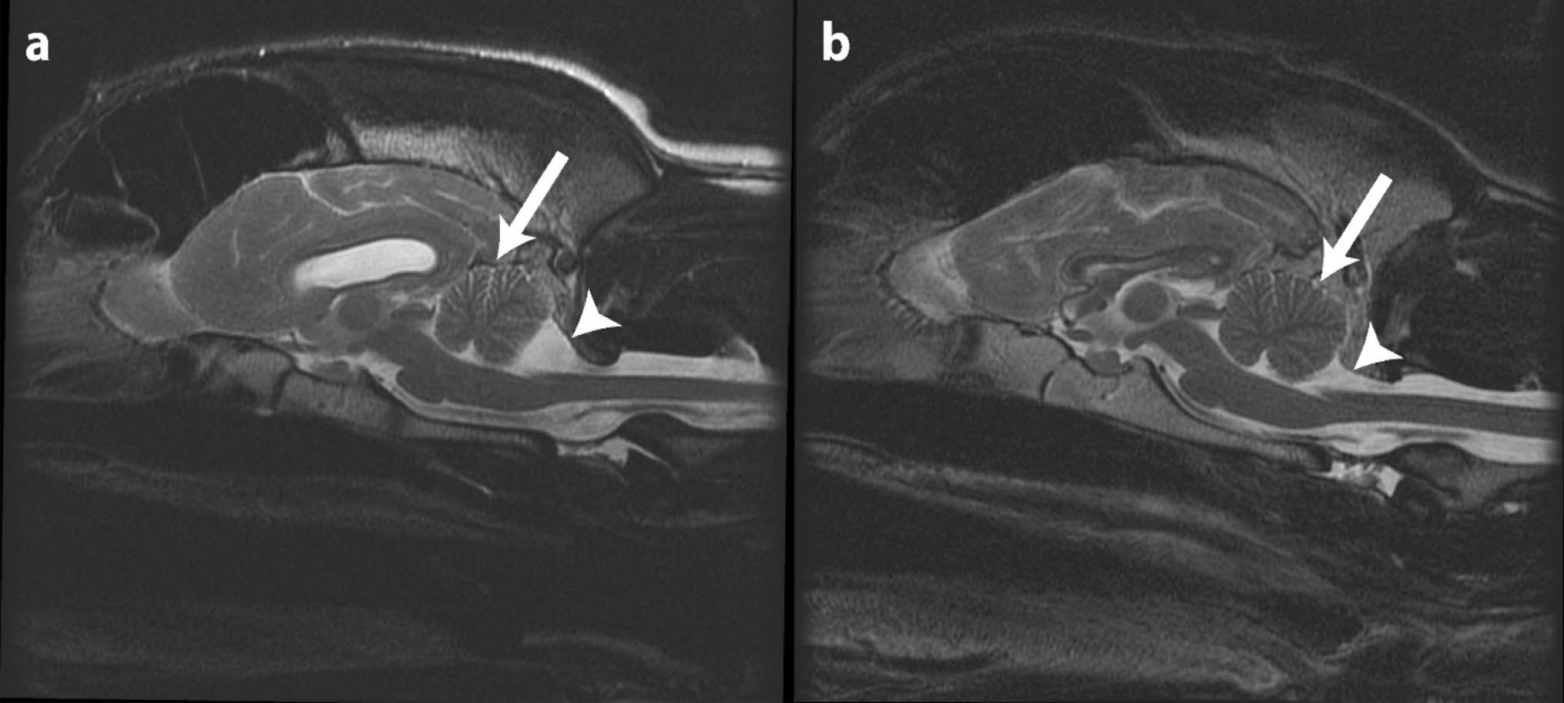
Comparison of T2 sagittal midline images of the brain of affected and non-affected Great Pyrenees. **a** T2 sagittal image of affected Great Pyrenees at 1 year of age. On midline cross-section, the cerebellum is small with increased fluid present between the folia (arrow). The caudal aspect of the cerebellum is flattened with a larger compensatory cerebellomedullary cistern (arrowhead). **b** Normal T2 sagittal image of the brain of a 3 year old Great Pyrenees. On midline cross-section, the cerebellum (arrow) is round and fills the dorsal portion of the caudal fossa. The cerebellomedullary cistern (arrowhead) is larger in this breed compared to many other breeds, but smaller than that of the affected dog

Survey radiographs of affected dogs did not reveal any abnormalities. Additional imaging studies (CT, with and without myelogram, *n* = 1; and MRI, *n* = 3) of the spine were generally inconclusive; only one MRI also included the head. In this case, the MRI was performed when the dog was 12 months of age, and demonstrated a smaller cerebellum (Fig. 2) and mild ventriculomegaly of the lateral ventricles.

### Disease description: pathological

**Fig. 3:**
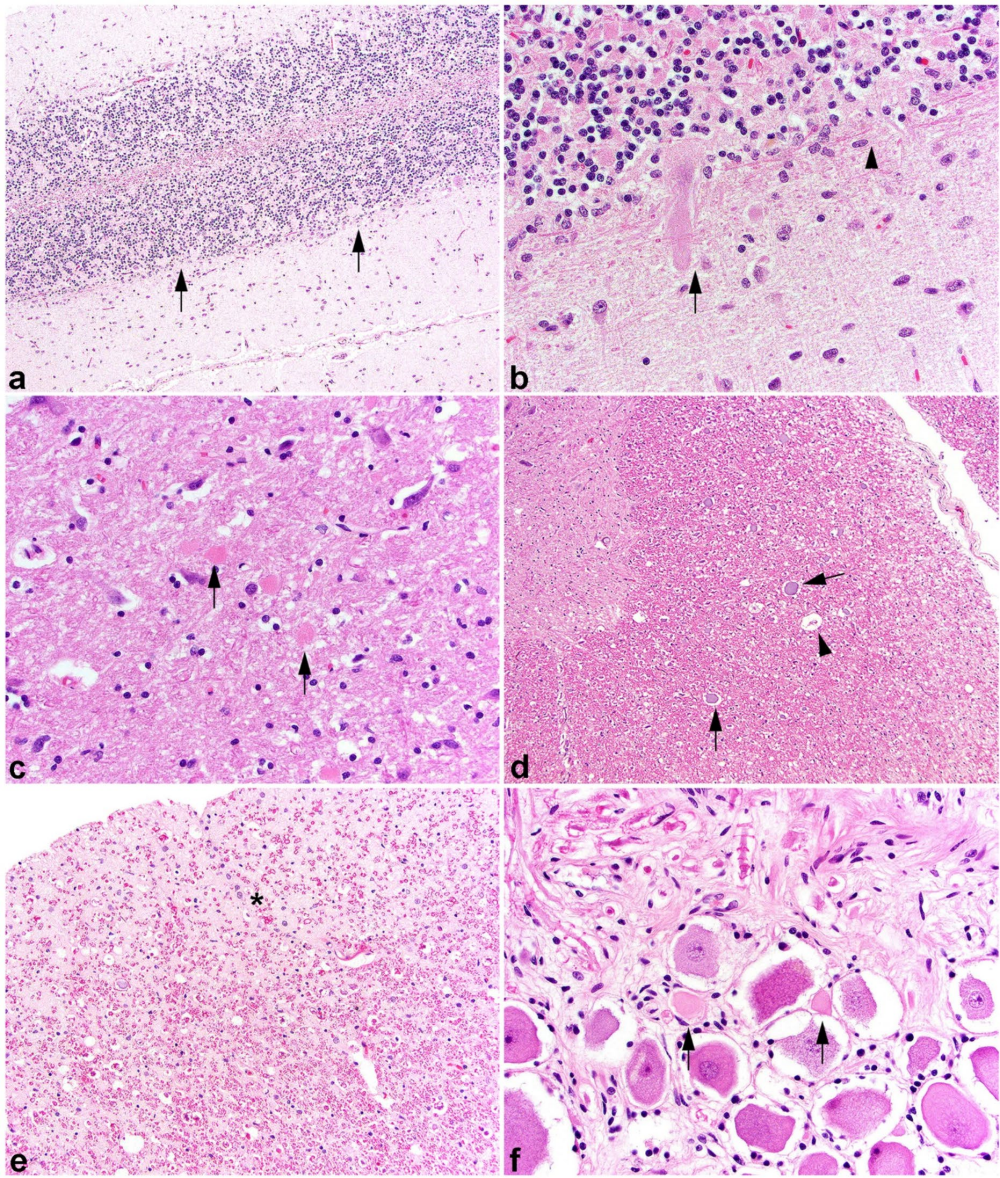
Central nervous system pathology of affected dogs. **a** Cerebellum, extensive loss of Purkinje cells. Arrows indicate regions completely devoid of Purkinje cells. Moderate gliosis replaces where the neurons have been lost. H&E stain, 100 × magnification. **b** Cerebellum, active degeneration found in some of the remaining Purkinje cells (arrow). The Purkinje cell layer is hypercellular consistent with Bergmann’s astrogliosis (arrowhead). H&E stain, 200 × magnification. **c** Necrotic neurons (arrows) are present in the medial geniculate nucleus. H&E stain. 200 × magnification. **d** Cervical spinal cord, marked white matter degeneration with spheroids (arrows) and myelin vacuolation with a digestion chamber (arrowhead). H&E stain,100 × magnification. **e** Cervical spinal cord, asterisk indicates dense astrogliosis replacing areas of marked white matter degeneration. H&E stain, 100 × magnification. **f** Degenerating ganglion cells with shrunken cell bodies, loss of Nissl substance, and hypereosinophilic cytoplasm (arrows). H&E stain, 200 × magnification

*Central nervous system* Necropsies were performed on seven of the affected dogs, however, a full histologic examination was only performed on four cases. Grossly, the cerebellum was obviously diminished in size, with an estimated decrease of one third to one-half of the normal cerebellum. No other gross lesions were noted. On histopathological examination, there was severe loss of cerebellar Purkinje cells throughout the cerebellar hemispheres (Fig. 3a), with the vermis generally less affected; there was concurrent degeneration and moderate astrocytosis (Bergmann astrocytosis) proliferating where Purkinje cells were lost in six of the seven dogs. One dog had an incomplete and sparse collection of central nervous system (CNS) tissues, rendering accurate assessment of the cerebellum and other CNS regions impossible. Active degeneration evidenced by hypereosinophilic cytoplasm, loss of nuclear detail, and irregular cell margins was found in remaining Purkinje cells (Fig. 3b). There was likewise severe degeneration and concomitant gliosis in the downstream cerebellar nuclei, where Purkinje cells project, as a result of Purkinje cell loss. Widespread neuronal degeneration/chromatolysis was observed in brainstem nuclei [including the medial vestibular, olivary, and medial geniculate (Fig. 3c)]. Scattered spheroids and myelin vacuolation were noted throughout white matter tracts in the brainstem. In all affected dogs in which the spinal cord was examined, there was significant white matter degeneration characterized by myelin vacuolation, digestion chambers, and scattered spheroids (Fig. 3d). In the most severely affected areas, marked astrogliosis in the dorsolateral and ventromedial funiculi and a lesser degree of degeneration in the dorsal funiculi was noted (Fig. 3e). Spinal gray matter had scattered chromatolytic and degenerate neurons in predominately the ventral horns.

*Peripheral nervous system* Skeletal muscles including the triceps brachii, vastus lateralis, and cranial tibial muscles, peripheral nerves including the peroneal and ulnar nerves, L4 and L5 dorsal nerve roots with portions of ganglia, and ventral nerve roots collected at necropsy from five affected dogs, and peripheral nerve and muscle collected from one dog following electrodiagnostic evaluation were evaluated. Pathological changes were similar in all dogs but varied in severity depending on progression of disease and the age of euthanasia.

Lumbar dorsal nerve root ganglia (DRG) in an affected dog euthanized at 7 years of age contained small numbers of degenerate ganglion neurons (Fig. 3f). While these can be observed rarely in older dogs, the number here was increased well beyond normal parameters. Specifically, dorsal root and ganglionic neurons and ventral roots had numerous inappropriately thin myelinated fibers found throughout. Occasional ganglionic neurons were also degenerating.

**Fig. 4:**
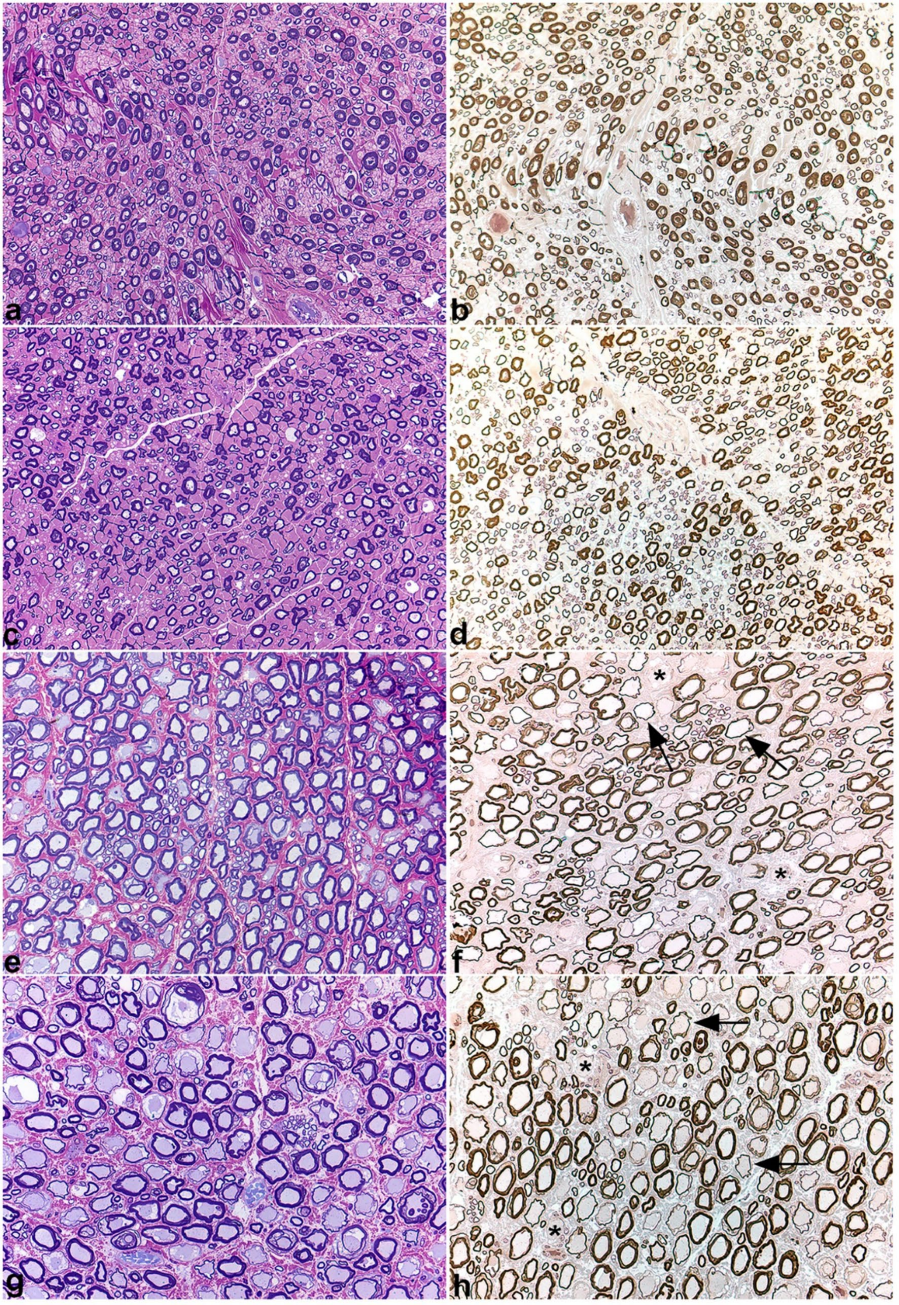
Peripheral nerve and nerve root histopathology. Resin sections of the peroneal (**a**, **b**) and ulnar (**c**, **d**) nerves, and dorsal (**e**, **f**) and ventral (**g**, **h**) nerve roots from a representative affected Great Pyrenees dog were cut (1 µm) and stained with toluidine blue basic fuchsin (**a**, **c**, **e**, **g**) or paraphenylenediamine (b, d, f, h). Large nerve fiber loss was apparent in the peroneal and ulnar nerves without obvious active axonal degeneration. Sporadic myelin ovoids were identified. Numerous inappropriately thin myelin sheaths for the axon diameter (arrows in f and h) were found in both the dorsal (f) and ventral (f) nerve roots. 40X magnification for all images

Skeletal muscles were either normal or showed a pattern of atrophy consistent with mild or early denervation. Fiber type grouping was not observed. Intramuscular nerve branches were normal in appearance. Large nerve fiber loss with a relative increase in smaller caliber nerve fibers, and occasional myelin ovoids were evident in all peripheral nerves evaluated without obvious axonal degeneration (Fig. 4a–d). Numerous inappropriately thin myelinated fibers for the axon diameters and demyelinated fibers with only thin myelin remnants were evident in both the dorsal and ventral nerve roots (Fig. 4e–h). Rare presumptive regenerating clusters were observed. Axonal degeneration was not obvious within the thinly myelinated fibers, however, small numbers of demyelinated fibers were degenerating. Occasional ganglionic neurons were also degenerating.

Of the eyes that were examined histopathologically (*n* = 3 dogs), ocular lesions were only noted in one dog; these consisted of frequent aggregates of fine, hazy, basophilic granules along the basement membrane beneath the retinal pigmented epithelium and along the processes of the photoreceptors (drusen). The optic nerve fiber was markedly thickened and vacuolated in some sections in this one affected dog.

### Genome-wide association mapping and haplotype analysis

Genome-wide SNP genotyping was performed on a discovery group, consisting of 32 dogs (those with red rim, Fig. 1), including 6 affected (2 females, 4 males) and 26 controls (16 females, 10 males). Standard chi-square association analysis did not highlight a single locus of interest; rather, multiple (*n* = 4, on CFA7, CFA19, CFA25, and CFA31) loci were observed, none of which achieved a raw p-value of less than 10^–4^. The genomic inflation factor (lambda) was only 1.2, indicating that the close pedigree family structure did not cause significant inflation to association test results and also that the relatedness among cases and among controls was very similar. Since a recessive mode of inheritance was suspected, the CFA7 locus was excluded because all cases were heterozygous at this locus; similarly, the CFA31 locus was excluded due to not all cases being homozygous. Next, a modified homozygosity mapping approach for five tests of association was undertaken using the --model command in PLINK. The top 100 SNPs (sorted by most significant p-value) are shown for all five tests in Supplemental Table S3. No locus perfectly conformed to the assumed fully penetrant autosomal recessive mode of inheritance, in which all six cases would be homozygous for that allele, with no control dog being homozygous for the allele. However, multiple SNPs overlapping with loci of interest CFA19 and CFA25 were identified as being homozygous in all six cases.

SNPs on CFA19 and CFA25 were phased to visually ascertain shared haplotypes among all cases. CFA19 was quickly ruled out due to the presence of multiple haplotypes among cases (not shown). Phasing the entirety of CFA25 (Supplemental Fig. 2) demonstrated that all cases were homozygous for the same haplotype from the start of CFA25 through the 27.74 Mb location (positions from CanFam3.1), containing over 150 genes. However, the CFA25 region highlighted by the dominant and genotypic tests in the modified homozygosity mapping approach stretched only 3.3 Mb in length, from positions 15.46–18.38 Mb. Furthermore, the two control dogs homozygous for the top CFA25 SNPs in the genotypic test were both found to have at least one haplotype that differed from the haplotype shared by all cases. A compelling positional and functional candidate gene, *sacsin molecular chaperone* (*SACS*, spastic ataxia of Charlevoix-Saguenay), positioned from 15.21 to 15.30 Mb, was located within the 27.74 Mb extended haplotype, and just upstream from the 3.3 Mb homozygosity-mapping haplotype (Supplemental Fig. 2).

### Identification and characterization of a *SACS* mutation

**Fig. 5:**
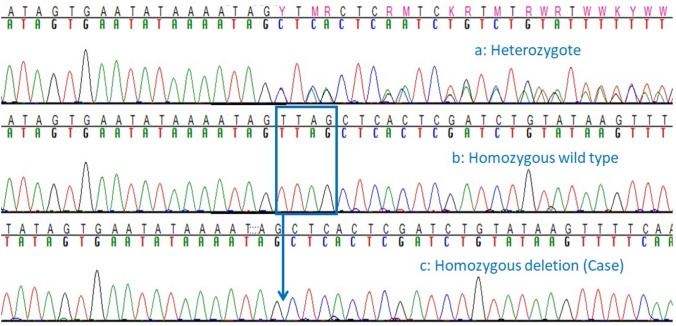
Chromatograms demonstrating 4pb *SACS* deletion. Sanger sequencing traces showing the *SACS* c.12731_12734delTTAG in: **a** heterozygous unaffected Great Pyrenees, **b** homozygous wild type unaffected Great Pyrenees, and **c** affected Great Pyrenees

We Sanger sequenced across all canine *SACS* exons (primers shown in Supplemental Table S2) in one affected dog. Seven coding variants were detected, three of which ultimately did not segregate with case/control status and three of which were synonymous changes. The remaining variant was a four bp deletion in the final exon (exon 10) (ENSCAFT00030020331.1:c.12731_12734delTTAG) detected in a homozygous state in this affected dog (Fig. 5). All additional known cases proved to be homozygous for the deletion, while five obligate carriers were heterozygous. Sanger sequence of 60 more controls were all homozygous wild type. The deletion causes a frameshift, comprising 31 miscoded amino acids, and resulting in a premature stop codon at the 32^nd^ post-deletion codon. This truncates 935 base pairs from the end of the last exon (exon 10, as well as any 3’ UTR), ultimately resulting in the loss of 343 amino acid residues, including the miscoded amino acids (p. Val4244AlafsTer32). The truncation of the protein deletes two entire functional domains: SacsJ (which binds chaperone HSP70) and HEPN (higher eukaryotes and prokaryotes nucleotide-binding); a portion of one sacsin internal repeat is also truncated from the protein structure. All dogs from the initial pedigree (Fig. 1) were genotyped, and the deletion variant perfectly segregated with case/control and obligate carrier status, confirming a recessive mode of inheritance.

### Post hoc linkage mapping and heritability calculations

A LOD (logarithm of the odds) score was calculated retrospectively using each dog’s *SACS* deletion genotype and the pedigree relationships shown in Fig. 1. A two-point autosomal linkage analysis generated a LOD score of 4.01 at a theta of zero, demonstrating significant linkage. The heritability of this condition derived from the genome-wide SNP array genotyped obtained on the pedigree dogs was estimated as 0.99999 (standard deviation of 0.68576E-03), confirming it as a single-gene, Mendelian condition.

### Great Pyrenees population study and breed specificity of *SACS* deletion

A random cohort of 133 Great Pyrenees dogs was genotyped to determine the prevalence of the deletion variant in the general breed population. A total of 125 were homozygous normal, 7 were heterozygous, and 1 was homozygous for the deletion (by chance, one of each genotype overlapped with the original discovery group), giving a carrier rate of 5.3%, a deletion allele frequency of 3.4%, and less than 1% of dogs in the Great Pyrenees breed population being affected. Subsequently, since the genetic test has been publicly available, a total of 972 Great Pyrenees have been genotyped, including both the original discovery group and the random population above. A total of 8 dogs were homozygous for the deletion (the 8 known cases), 106 were heterozygous, and 858 were homozygous normal, giving a carrier rate of 10.9%, a deletion allele frequency of 6.3%, and 0.8% of dogs affected. This latter group represents a significant ascertainment bias, as dogs related to cases were preferentially tested, and more likely to be carriers. Lastly, we examined whole genome sequences available from over 1300 dogs representing 126 breeds and several wolves (Jagannathan et al. 2019; Plassais et al. 2019) for the 4 bp *SACS* deletion and found that all dogs were homozygous for the reference sequence.

## Discussion

Here, we describe a novel spontaneously occurring canine genetic model of ARSACS that is perfectly associated with homozygosity for a 4 bp deletion variant in the *SACS* gene. ARSACS is one of a large, genetically and clinically heterogeneous group of recessive spinocerebellar ataxias (Bereznyakova and Dupré 2018; Fogel 2018; Synofzik and Németh 2018). First described in a founder population in Quebec, Canada, but subsequently reported worldwide, often in consanguineous lineages, ARSACS is now recognized as one of the most important causes of autosomal recessive ataxias (Parkinson et al. 2014). This neurodegenerative disease is a childhood-onset condition characterized by cerebellar ataxia, pyramidal spasticity, and peripheral neuropathy; the progressive degeneration of the cerebellum and spinal cord is associated with progressive loss of Purkinje neurons (Bouhlal et al. 2011; Girard et al. 2012). Clinically, classic ARSACS patients exhibit early-onset spasticity in the lower limbs manifesting as difficulty in walking and gait unsteadiness as early as infancy, but typically between 1 and 2 years of age (Bouhlal et al. 2011). Cerebellar signs are sparse at the beginning of the ARSACS disease course, but increase slowly over time; eventually motor axonal polyneuropathy aggravates the deficits (Bereznyakova and Dupré 2018). The disease course is long, and ARSACS patients become wheelchair-bound, on average by their early 40s (Bouchard et al. 1978). *SACS*^−/−^ mice also had abnormal gaits with progressive motor, cerebellar, and peripheral nerve dysfunctions (Larivière et al. 2015). The Great Pyrenees cases described in this study fit closely with the typical early-onset phenotype, as pups’ gaits were noticeably abnormal as early as 3–4 months, and likewise exhibited slowly progressive disease culminating in severe paresis of the pelvic limbs by middle age. The early age of onset observed in affected dogs homozygous for the protein-truncating deletion is consistent with what is observed in humans, where the age of onset is mainly affected by the pathogenicity of the mutation (Xiromerisiou et al. 2020). The progression from cerebellar signs predominating at younger ages to neuromuscular weakness being more pronounced later in disease progression in affected dogs is very similar to the progression seen in the degenerative myelopathy (*SOD1* mutation, similar to human amyotrophic lateral sclerosis) in Boxers and Pembroke Welsh Corgis (Shelton et al. 2012). Such progression may therefore not be unusual with CNS degenerative diseases in dogs.

A variable feature in ARSACS patients is the degree of mental impairment, which can range from absent to mild/moderate (Bereznyakova and Dupré 2018); in fact, it is estimated that approximately half of ARSACS patients have some level of intellectual disability (Pilliod et al. 2015). This feature is difficult to assess in dogs, however, owners reported no perceivable deficiencies in affected dogs’ mentation.

The prominent demarcation of retinal nerve fibers radiating from the optic disc in ARSACS patients were formerly designated yellow streaks of hypermyelinated fibers (as visible on fundoscopic exam), but are now considered retinal nerve fiber hypertrophy (Pablo et al. 2011; Garcia-Martin et al. 2013a, b), measurable via OCT. The increase in thickness is thought to be caused either by hypertrophy or an augmented number of retinal nerve fibers (Bouchard et al. 1978; Seibold et al. 2010). While it was always known as one of the main clinical manifestations of ARSACS in the French-Canadian population, this feature has seldom been reported in non-Quebec patients (Bouhlal et al. 2011; Bereznyakova and Dupré 2018). Further, there is variability of this phenotype even within families harboring the same mutation (Bereznyakova and Dupré 2018). Failure to identify this ocular abnormality in affected Great Pyrenees fits with the inconsistency of this feature in ARSACS patients; possibly, this variability is related to the location of the deletion mutation within the gene, or due to epigenetic and/or environmental factors. Despite the fact that affected dogs did not undergo OCT, it is unlikely that this phenotype was missed on fundoscopic exam in the affected dogs, because this change is observed via typical fundoscopic examination in 89% of affected human ARSACS patients (Xiromerisiou et al. 2020). However, it should be noted that the degree of myelination of the canine optic nerve head varies considerably between individual animals, so a subtle change might not be easy to detect over normal variation. Nystagmus is a common feature of ARSACS patients, in either a constant state (El Euch-Fayache et al. 2003) or gaze-evoked (Hara et al. 2005; Parkinson et al. 2014; van Lint et al. 2016). Subtle nystagmus was only reported in one affected Great Pyrenees, and this nystagmus was not observed during neurologic evaluations.

Motor nerve conduction velocities are decreased in ARSACS patients (Parkinson et al. 2014), with a mixed demyelinating and axonal character (Baets et al. 2010), indicating axonal involvement and peripheral myelinopathy (El Euch-Fayache et al. 2003). Decreased motor nerve conduction velocities were likewise observed in the one affected Great Pyrenees where this was measured. The absence of spontaneous activity in muscles by electromyography, the normal amplitude of the compound muscle action potential, and the markedly decreased motor nerve conduction velocity would be consistent with a primary demyelinating polyneuropathy with secondary axonal degeneration as nerve fibers become fully demyelinated. The electrodiagnostic findings are also consistent with the minimal to absent pattern of denervation atrophy within the muscles, and normal intramuscular nerve branches.

Peripheral nerve biopsies of human ARSACS patients reveal a pattern consistent across patients of all ethnicities: severe axonal degeneration with loss of large myelinated fibers associated with variable axonal degeneration and regenerating axonal clusters, thinning of myelin sheaths, rare demyelinating aspects, and rare onion bulbs (Peyronnard et al. 1979; El Euch-Fayache et al. 2003; Takiyama 2006; Bouhlal et al. 2011). Skeletal muscle biopsies appear typical for neurogenic atrophy (Parkinson et al. 2014). In contrast, in the knock-out mouse model no obvious axonal degeneration or demyelination was found in mid-thigh segments of sciatic nerves but a significant decrease in the number of large myelinated axons and a relative increase in small caliber axons were found. Peripheral nerve biopsies were similar in both knock-out mice (Larivière et al. 2015) and in the affected Great Pyrenees but differ from that of human cases. These species differences could be a result of the long duration of clinical diseases in people (decades) versus 4–5 years in dogs, and months in mice.

Atrophy of the superior cerebellar vermis is always present on MRI in ARSACS patients (Dupré et al. 2006; Bereznyakova and Dupré 2018), and this was similarly observed in the one affected dog who underwent MRI of the head (Fig. 2). This patient did not undergo additional imaging, but based on the gross appearance of the cerebellum at autopsy, the cerebellar atrophy continued to progress until euthanasia.

Autopsies of ARSACS patients demonstrate a grossly atrophied superior cerebellar vermis (Bouchard et al. 1998); likewise, in autopsied affected Great Pyrenees, there was a gross decrease in cerebellar size. Post-mortem histopathological examinations in human ARSACS patients reveal features including neuronal lipid storage and absence of Purkinje cells in the cerebellum (Bouchard et al. 1998; Ali et al. 2016). The loss of cerebellar Purkinje cells has been reproduced in the sacsin-depleted mouse model of ARSACS; specifically, the *SACS* knock-out mouse demonstrates an age-dependent loss of Purkinje cells, and this is consistent with the observed progressive ataxia (Girard et al. 2012; Larivière et al. 2015). Purkinje cell loss in affected Great Pyrenees mimicked these findings, with severe Purkinje cell loss throughout the cerebellar hemispheres. This loss worsened with age, as the older dogs had very few Purkinje cells left. The pathological changes to the DRG appear unique to the canine syndrome and indicate variation in phenotype between the human disease, knock-out mouse model, and canine patients. Taken together, the lesions from all canine cases indicate widespread multisystem neurodegenerative syndrome, with nuclei affected in multiple different CNS systems.

The histopathological ocular lesions in the single dog were likely unrelated to this neurological syndrome, as the eyes from two other dogs, both euthanized a much older ages with more significant neurological disease progression, had no lesions. Furthermore, clinical ocular (fundoscopic) examination of two dogs by a veterinary ophthalmologist revealed only subtle changes.

Human ARSACS is caused by variants in the *SACS* gene (Engert et al. 2000), the last exon of which is the largest known vertebrate exon, spanning nearly 12 kb. The 4 bp canine deletion described here is located in this final exon, where the majority of human *SACS* mutations have been observed (Xiromerisiou et al. 2020). *SACS* codes for the extremely large sacsin protein (Engert et al. 2000; Parfitt et al. 2009), which at 4579 residues in length (520 kDA) is one of the largest proteins encoded by the human genome (Bradshaw et al. 2015) (GenBank NP_055178.3). Canine sacsin is equivalently large, consisting of 4587 residues (ENSCAFT00030020331.1). Sacsin is expressed ubiquitously in the mammalian CNS, with very high expression in the brain, particularly in the brain motor systems; the highest levels are in large neurons, such as cerebellar Purkinje cells (Parfitt et al. 2009; Bouhlal et al. 2011; Bereznyakova and Dupré 2018). Sacsin is also present in skeletal muscles and fibroblasts (Engert et al. 2000).

Sacsin is a multi-modular protein, composed of the following functional domains: an N-terminal ubiquitin-like domain that interacts with the proteasome; three large chaperone domains homologous to HSP90 in repeated specific internal regions (called sacsin internal repeats, SIRPTs; functionally important for regulating protein folding); a sacsin J-domain (SacsJ) that binds chaperone HSP70; and a C-terminal higher eukaryotes and prokaryotes nucleotide-binding (HEPN) domain that mediates sacsin dimerization and the sacsin repeating regions (Grynberg et al. 2003; Parfitt et al. 2009; Anderson et al. 2010, 2011; Kozlov et al. 2011; Pilliod et al. 2015; Gentil et al. 2019). Based on the 3’ location of the 4 bp deletion within the canine *SACS* gene, and subsequent premature stop codon and truncation of the sacsin protein, affected dogs will lose all of the SacsJ and HEPN functional domains, as well as approximately half of the final (third) SIRPT. The loss of the SacsJ domain, in particular, supports the idea that sacsin will have lost critical chaperone functions, both in ARSACS cases (Dupré et al. 2006; Bouhlal et al. 2011) and the affected dogs in this study. This is confirmed by the fact that human ARSACS patients with mutations affecting the C-terminal region of the protein have a similarly severe clinical phenotype (Bouhlal et al. 2011). Interestingly, clinical and pathological phenotypes comparable to those observed in *Sacs*^*−/−*^ mice, were observed in a recently generated *Sacs*^*R272C*^ mouse, where mice homozygous for this missense-mutation still maintained some sacsin expression (Larivière et al. 2019). This indicates that even missense *SACS* mutations affecting the N terminal region of the protein are likely to interfere with sacsin function, and strongly demonstrates that deletion-frameshift-truncation mutations such as that described in the canine model are highly pathogenic.

The physiologic role of sacsin is still being actively investigated, but the presence of chaperone and UBL domains suggest protein quality control/proteostasis functions (Parfitt et al. 2009; Duncan et al. 2017; Fogel 2018; Synofzik and Németh 2018). Recent work demonstrates sacsin’s role in regulating assembly and dynamics of intermediate filaments (Gentil et al. 2019); pathogenic mechanisms of abnormal sacsin change the organization of the intermediate filament cytoskeleton, and result in accumulation and bundling of perikaryal and dendritic intermediate neurofilaments (Duncan et al. 2017; Gentil et al. 2019). Intermediate filaments also have a known role in intracellular organelle distribution (Tang et al. 2008; Gentil et al. 2012). In the *Sacs*^*−/−*^ mouse, the abnormal neurofilament accumulations in somatodendritic regions of neurons occurred as early as 2 weeks after birth (Larivière et al. 2015), which suggests that cytoskeletal network alterations are key early cellular dysfunctional components in human ARSACS and the present canine patients. Loss of sacsin in *Sacs*^*−/−*^ mice also alters mitochondrial morphology, dynamics, and distribution, collectively resulting in impaired oxidative phosphorylation and increased oxidative stress (increased production of reactive oxygen species) (Bradshaw et al. 2015; Criscuolo et al. 2015), corroborating the relationship between mitochondria, oxidative stress, and neurodegeneration (Gandhi and Abramov 2012). Furthermore, the problems with the intermediate filament cytoskeleton and mitochondrial function are not mutually exclusive, given the importance of the cytoskeleton in regulating mitochondrial dynamics (Anesti and Scorrano 2006; Chen and Chan 2009).

There is no curative treatment for ARSACS; symptomatic management is used for spasticity and various therapies (physical, occupational, speech language, etc.) are commonly utilized (Larivière et al. 2015; Bereznyakova and Dupré 2018). Affected dogs in the present study received treatments ranging from none, to steroids, to physical therapies with cart assistance. Affected dogs had a shortened lifespan, and early euthanasia was often chosen. This deletion variant is now offered as a genetic test to Great Pyrenees breeders for use in mating decisions. With the availability of a genetic test, dog breeders and owners can now screen their breeding stock and avoid producing affected puppies by not breeding carriers (dogs heterozygous for the deletion) to one another.

In conclusion, the clinical and pathological picture presented by the Great Pyrenees affected with the neurodegenerative disorder in the present study very closely mimics those seen in human ARSACS. Molecular genetic studies demonstrated these dogs have a deleterious *SACS* mutation. The clinical phenotype is very highly heritable, and the discovered variant appears to be private to the Great Pyrenees breed. Thus, we confirm the first ever naturally occurring, and large animal, model of human ARSACS.

### Supplementary Information

Below is the link to the electronic supplementary material.

Supplemental Table 1 (DOCX 43 KB)

Supplemental Table 2 (DOCX 37 KB)

Supplemental Table 3: Results of modified homozygosity mapping approach, using PLINK - - model test, with - - fisher command, on Great Pyrenees discovery group (n = 32). The top 100 SNPs are shown for each test, sorted by p-value. Allele counts are provided for each of the five tests, separated into cases (“Aff”) and controls (“Unaff”). The homozygous CFA25 LD region is bolded and is best observed in the “Geno” and “Dom” tests. The CFA25 region was not detected in the “Rec” test, despite the condition clearly being autosomal recessive. This seems counterintuitive, until recognizing that the variant allele, and its tagging haplotype, were actually more common in our discovery group compared to the reference/normal allele, because we had so many carrier (heterozygous) dogs. This resulted in PLINK considering the variant allele “dominant”, simply because it was the major allele (PLINK designates the minor allele “recessive”)

Supplemental Figure 1 (DOCX 506 KB)

Supplemental Figure 2: Phased haplotypes across all of CFA25. Data from each dog occupies two rows, one for each chromosome. Six cases are shown in red, and four unaffected parents (obligate carriers) in purple. Remaining dogs are also unaffected. Each SNP represents one column; SNP IDs and bp positions are provided at the top; CanFam3.1 positions are used. All cases were homozygous for a shared haplotype from the start of CFA25 through 27.74 Mb. The region identified through dominant and genotypic tests in PLINK covers 3.3 Mb from 15.46 Mb to 18.38 Mb. The most significant SNPs, all in LD with one another, are highlighted in blue. SACS’s location is shown in orange

## Data Availability

The discovery group canine SNP data has been deposited in Dryad (https://datadryad.org), and is publicly available under the DOI: https://doi.org/10.5061/dryad.ht76hdrn5.
