# Supplementary material for: A SACS deletion variant in Great Pyrenees dogs causes autosomal recessive neuronal degeneration
Source: Hum Genet. 2023 Sep 27;142(11):1587–601. doi: 10.1007/s00439-023-02599-1 (PMC10602964; doi:10.1007/s00439-023-02599-1)
Supplement: Supplementary file 1 — Supplemental Table 1 (DOCX 43 KB) [file 439_2023_2599_MOESM1_ESM.docx]

**Supplemental Table 1: Primers used for Sanger sequencing of all canine *SACS* exons.** Primers were built using ENSCAFT00030020331.1 canine sequence from Ensembl ([www.ensembl.org](http://www.ensembl.org)) and primers were designed using Primer3 (http//bioinfo.ut.ee/primer3/) (Untergasser et al. 2012).
