# Supplementary material for: A SACS deletion variant in Great Pyrenees dogs causes autosomal recessive neuronal degeneration
Source: Hum Genet. 2023 Sep 27;142(11):1587–601. doi: 10.1007/s00439-023-02599-1 (PMC10602964; doi:10.1007/s00439-023-02599-1)
Supplement: Supplementary file 2 — Supplemental Table 2 (DOCX 37 KB) [file 439_2023_2599_MOESM2_ESM.docx]

| **S2 Table: Allele-Specific PCR for *SACS*-Associated NDG Variant** | |
| --- | --- |
| Primer Name | Primer Sequence |
| **NDG-SACS F** | CAATACCTGCCGAAATCCAT |
| **NDG-SACS N R** | CAGATCGAGTGAGCTAACT |
| **NDG-SACS A R** | ATACAGATCGAGTGAGCTATT |
| **LPN1-ARHGEF10 F** | AGCCACTTTCGGGATTCTTC |
| **LPN1-ARHGEF10 R** | TGTTCCCTTGGTCACAGGAC |

**Supplemental Table 2: Primers used in genetic testing for detection of *SACS* c.12731_12734delTTAG normal and deletion alleles via an allele-specific PCR.** There are two reverse primers: N (normal) and A (affected). Primers for the internal control (designated LPN1) are also listed; this product is located on CFA16.
