# Supplementary material for: A SACS deletion variant in Great Pyrenees dogs causes autosomal recessive neuronal degeneration
Source: Hum Genet. 2023 Sep 27;142(11):1587–601. doi: 10.1007/s00439-023-02599-1 (PMC10602964; doi:10.1007/s00439-023-02599-1)
Supplement: Supplementary file 4 — Supplemental Figure 1 (DOCX 506 KB) [file 439_2023_2599_MOESM4_ESM.docx]

**
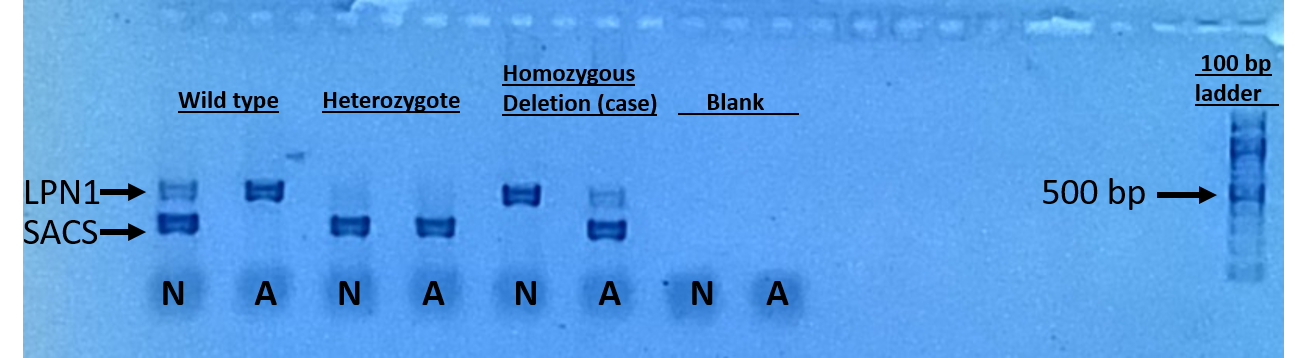
**

**Supplemental Figure 1: Agarose Gel Image of Allele-Specific Genotyping Assay.** Each *SACS*-deletion genotype is demonstrated (n = 3 dogs): homozygous wild type, heterozygous, and homozygous deletion. Each dog is tested twice, once with each *SACS* reverse primer (designated N or A, see **Supplemental Table 2**). Every reaction includes an internal control pair of primers, designated LPN1, on a different chromosome (CFA16). The LPN1 product is larger, so it will amplify less or not at all when the other allele is present.
